# Simple Rule for Ascertaining the Length of Days and Nights

**Published:** 1897-12

**Authors:** 


By a simple rule the length of the day and night, any time of the year, may be ascertained by simply doubling the time of the sun's rising, which will give the length of the night, and double the time of the setting will give the length of the day.—*Exchange*

